# Oleoylethanolamide Modulates BDNF-ERK Signaling and Neurogenesis in the Hippocampi of Rats Exposed to Δ^9^-THC and Ethanol Binge Drinking During Adolescence

**DOI:** 10.3389/fnmol.2019.00096

**Published:** 2019-04-24

**Authors:** Daniel Silva-Peña, Patricia Rivera, Francisco Alén, Antonio Vargas, Leticia Rubio, Nuria García-Marchena, Francisco Javier Pavón, Antonia Serrano, Fernando Rodríguez de Fonseca, Juan Suárez

**Affiliations:** ^1^Instituto de Investigación Biomédica de Málaga, U.G.C. de Salud Mental, Hospital Regional Universitario de Málaga, Málaga, Spain; ^2^Department of Endocrinology, Fundación Investigación Biomédica del Hospital Infantil Universitario Niño Jesús, Madrid, Spain; ^3^Departamento de Psicobiología, Universidad Complutense Madrid, Pozuelo de Alarcón, Spain; ^4^Departamento de Anatomía y Medicina Legal, Universidad de Málaga, Málaga, Spain

**Keywords:** alcohol, brain-derived neurotrophic factor, ERK, hippocampus, memory, oleoylethanolamide

## Abstract

Oleoylethanolamide is an endogenous NAE that modulates ethanol-seeking behavior and ethanol-induced neuroinflammation. In the present study we further analyze the role of OEA in hippocampal neurogenesis, BDNF-ERK signaling, and spatial memory that are affected by alcohol. Additionally, we addressed the effects of OEA on the association of alcohol and cannabis, a frequent combination in human alcohol addicts, and whose long-term effects are far from being understood. To this end, OEA (10 mg/kg/day, i.p.) was pharmacologically administered for 5 days/week in a preclinical model of adolescent rats with binge-like consumption (1 day/week) of ethanol (3 g/kg, i.g.) combined or not with acute administrations of Δ^9^-THC (5 mg/kg, i.p.) for 5 weeks. OEA restored ethanol/THC-related decreases in both short-term spatial memory (spontaneous alternation by Y-maze) and circulating levels of BDNF, reduced cell proliferation (*Mki67* and IdU+ cells) and maturation (*Dcx*, *Calb1*), and improved cell survival (*Casp3* and BrdU+ cells) in the dorsal hippocampus. Interestingly, OEA alone or combined with THC also decreased the mRNA levels of neurotrophic factors (*Bdnf*, *Ntf3*) and the NT3 receptor *TrkC*, but increased the BDNF receptor *TrkB* in the hippocampus of ethanol-exposed rats. These effects were likely associated with a OEA-specific phosphorylation of AKT and ERK1, key signaling regulators of cell proliferation and survival. These results suggest a regulatory role of OEA in short-term spatial memory and hippocampal neurogenesis through BDNF/AKT/ERK1 signaling in response to acute THC in an alcoholic context during adolescence.

## Introduction

The excessive consumption of alcohol is associated with alterations in brain physiology, structure and function. The effects of alcohol are concentration-dependent and the patterns of excessive alcohol intake, such as binge drinking, are becoming predominant, especially in young drinkers. Alcohol binge drinking is defined as the pattern of consumption that increases the blood alcohol concentration above 80 mg/dL, which occurs after a consumption of about 56–70 g of pure alcohol in ≤2 h (NIAAA, 2004; Pilatti et al., 2017). At early ages, exposure to alcohol binges increases subsequent alcohol intake during adolescence, but not adulthood (Fabio et al., 2014). Alcohol intake initiation in binge episodes may induce long-lasting consequences in the adult brain and increases lifetime risks of developing adult psychopathology, particularly alcohol use disorders (AUD) (Crews et al., 2016). Alcohol-induced neurodegeneration is associated with changes in neuronal processes such as apoptosis and neuronal proliferation and maturation (Nixon and Crews, 2004).

A relevant issue when considering the impact of alcohol abuse on brain development and function is its association with the consumption of additional psychoactive drugs, mainly cannabis. It is well-known that Δ^9^-tetrahydrocannabinol (THC), the primary psychoactive component of cannabis, has an impact on brain development and function, and short term-memory deficits induced by THC have been well documented (Iversen, 2003). A potential brain area where alcohol and cannabis might converge is the hippocampus, a place notably affected by acute and chronic alcohol exposure. The mammalian hippocampus is a brain region strongly related to memory processes and adult neurogenesis (Moreno-Jiménez et al., 2019). It contains a high density of cannabinoid CB1 receptors (Rivera et al., 2014), the main target of THC, suggesting that this area is an important locus for cannabinoid effects on learning and memory (Varvel and Lichtman, 2005). Both acute and chronic exposure to cannabis are associated with dose-related cognitive impairments, affecting different cognitive areas, especially attention, working memory, verbal learning, and memory functions (Zanettini et al., 2011) and humans (Solowij and Battisti, 2008).

Several endogenous mechanisms capable of counteracting alcohol-induced damage have been identified. Among them, OEA is a promising lipid transmitter capable of counteracting both alcohol-induced neuroinflammation (Antón et al., 2017) and alcohol seeking behavior (Bilbao et al., 2016). OEA belongs to a NAE family involved in the regulation of multiple physiological functions such as feeding behavior, addiction, and cancer cell proliferation (Sihag and Jones, 2018; Zhao et al., 2018). The roles of OEA are mediated primary by the peroxisome proliferator-activated receptor alpha (Orio et al., 2013) and other targets such as the transient receptor potential vanilloid type-1 or the orphan G protein-coupled receptors 119 and 55 (Antón et al., 2017). Recent preclinical evidence indicated that OEA is involved in homeostatic protective mechanisms in response to alcohol abuse (Bilbao et al., 2016). OEA is released after alcohol administration in rodents, and its exogenous administration regulates alcohol relapse and reduces several withdrawal symptoms of alcohol (Bilbao et al., 2016; Antón et al., 2017). A recent study of our group described that plasma concentrations of OEA are increased in abstinent patients diagnosed with AUD, suggesting that OEA may act as a potential biomarker for predicting length of alcohol abstinence (García-Marchena et al., 2017a). Neuroprotective and anti-inflammatory profiles of OEA are not restricted to alcohol, but also demonstrated in animal models of neurological disorders, such as Parkinson’s disease or brain ischemia (Zhou et al., 2012; Gonzalez-Aparicio et al., 2014).

Neurotrophic factors, specially the BDNF, constitute another endogenous mechanism involved in alcohol effects (Silva-Peña et al., 2018). Neurotrophic factors are a family of peptides and small proteins involved in a variety of adaptive functions in the developing and mature brain, particularly neuronal growth, differentiation, survival, synaptic plasticity, and the formation of long-lasting memories and behavioral consolidation (Lu et al., 2014). BDNF mainly binds to the high-affinity receptor tyrosine kinase tropomyosin-related kinase B (TrkB) and initiates an intracellular downstream signaling, thereby recruiting transcriptional and translational mechanisms via the MAP kinase ERK1/2 and phosphoinositol 3-kinase (PI3K) pathways (Huang and Reichardt, 2003). BDNF-TrkB signaling system and ERK downstream effectors on transcription are highly implicated in cell cycle, cell phenotype decisions, postmitotic activities, and synaptic plasticity (Bodart, 2010; Roskoski, 2012). These processes are highly involved in the formation of learning and memory in the hippocampus (Andero et al., 2014). Optimal functioning of the BDNF-TrkB-ERK signaling may be a susceptibility factor for developing and maintaining poor recovery from impaired memory and altered hippocampal functions. Recent clinical studies have shown that circulating levels of BDNF are decreased in abstinent patients diagnosed with AUD (García-Marchena et al., 2017b). Moreover, reduced BDNF levels in the plasma of alcohol-dependent patients correlated with severe scores of cognition deficits, whereas chronic consumption and reinstatement of ethanol in adolescent rats was associated with lower plasma levels of BDNF, as well as with decreases in *Bdnf* mRNA levels, phosphorylated ERK2 levels and neurogenic responses (*Mki67*, *Sox2*, *Dcx*, *Ncam1*, and *Calb1*) in the hippocampus (Silva-Peña et al., 2018). These neurogenesis-related factors are implicated in the correct hippocampal functioning like cellular proliferation, maintenance of neural stem cells and neuronal precursor cells, and neurite outgrowth including humans (Knoth et al., 2010; Moreno-Jiménez et al., 2019). Besides, a negative correlation between hippocampal *Bdnf* mRNA levels and recognition memory was found under an alcoholic context (Silva-Peña et al., 2018).

Thus, here the main hypothesis under test was that OEA regulates BDNF signaling in a model of adolescent rats exposed to ethanol and THC. The homeostatic role for OEA has not been still explored in hippocampal functioning related to neurogenesis, BDNF-ERK signaling and memory that become notably altered by alcohol and partially affected by cannabinoid activation. Using a multi-disciplinary approach and a pharmacological administration of OEA (10 mg/kg/day, i.p.) for 5 days/week in a rat model of binge-like consumption (1 day/week) of ethanol (3 g/kg, i.g.) together with acute administrations of Δ^9^-THC (5 mg/kg, i.p.) for 5 weeks during adolescence, we explored the effects of OEA treatment on (1) spatial memory-like behavior, (2) mRNA expression of components of the neurotrophic –BDNF/NT3– system, (3) phosphorylation of intracellular protein kinases (AKT, ERK), and (4) cell proliferation, maturation, and survival in the dorsal hippocampus.

## Materials and Methods

### Ethics Statement

Experiments and procedures were conducted under strict adherence to the European Directive 2010/63/EU on the protection of animals used for scientific purposes and with Spanish regulations (RD 53/2013 and 178/2004). All efforts were made to minimize unnecessary suffering. All protocols were approved by the Ethics and Research Committee of Universidad de Málaga (CEUMA, 7-2016-A).

### Subjects

Animal studies were conducted on 5 to 10-weeks-old male Wistar rats (Charles River Laboratories, Barcelona, Spain) weighing ∼100 g at the beginning of the experiments. Rats were individually housed in clear plastic cages in a vivarium under standard controlled conditions: a 12-hour light/dark cycle (lights off at 8:00 pm.), ambient temperature (21 ± 2°C) and humidity (65 ± 5%); at the Animal House Centers of University of Málaga (Spain). Unless otherwise indicated, tap water and food (Purina chow) were available *ad libitum* throughout the course of these studies.

### Ethanol Binge Exposure

**FIGURE 1:**
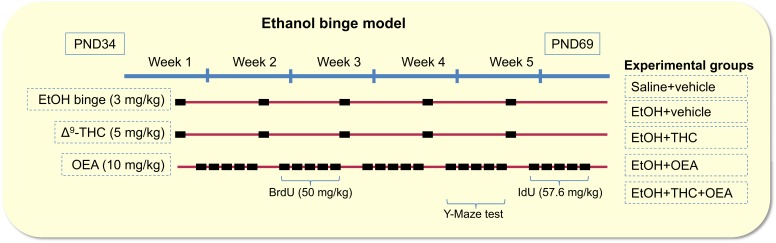
Time line of the experimental design for a rat model of binges-like orally ethanol consumption (3 g/kg, 1 day/week) together with i.p. administration of THC (5 mg/kg, 1 day/week) and i.p. administration of OEA (10 mg/kg, 5 days/week) for 5 weeks during adolescence (PND34-69). The experiment also included BrdU (50 mg/kg) and IdU (57.6 mg/kg) administrations for 5 days after week 2 and 5, respectively, and the behavioral Y-maze test in week five.

This experimental model was designed to mimic the 1-day heavy drinking of the adolescent population. The experimental model and treatments were performed twice to constitute two batches. Young rats (*n* = 80) were firstly habituated to the experimental conditions, including handling and injection procedures (holding and pseudo-injection) during one week before the experimentation in order to minimize stress effects (Sánchez-Marín et al., 2017). Then, from PND34 to PND69, four groups of rats were weekly (on Friday) exposed to single binge intragastric (i.g.) administrations of 25% (*v*/*v*) ethanol (3 g/kg) for five consecutive weeks (Figure 1).

### Drug Administration

Three groups of ethanol binge-exposed rats were treated with the principal psychoactive constituent of cannabis Δ^9^-tetrahydrocannabinol (THC or dronabinol; THC Pharm GmbH, Frankfurt am Main, Germany) and/or the endogenous PPARα receptor agonist oleoylethanolamide (OEA; cat. no. 1484, Tocris, Abingdon, United Kingdom). THC were dissolved in a solution containing 25% (*v*/*v*) ethanol in sterile 0.9% NaCl solution. OEA were dissolved in a vehicle containing 10% (*v*/*v*) Tween80 in sterile 0.9% NaCl solution. Both drugs were prepared just before each administration, and they were intraperitoneally (i.p.) injected in a final volume of 1 mL/kg of body weight. The optimal dose at which treatment would be more effective in behavior and neuroprotection, as described previously (Piomelli et al., 2006; Galan-Rodriguez et al., 2009; Murphy et al., 2017), was selected for the present study. Two groups of ethanol binge-exposed rats were treated with 5 mg/kg of THC once per week (on Friday), just before ethanol i.g. administration, for five consecutive weeks. Two groups of ethanol binge-exposed rats were treated with 10 mg/kg of OEA, 48 h after THC and/or ethanol administrations, 5 days per week (from Sunday to Thursday) for five consecutive weeks. Thus, total sample (*n* = 40 per batch, *n* = 8/group) was divided into five groups (Figure 1): Saline-Vehicle, Ethanol-Vehicle, Ethanol-OEA, Ethanol-THC, and Ethanol-OEA-THC.

### BrdU and IdU Administration

5-bromo-2′-deoxyuridine (BrdU; cat. no. B5002, Sigma-Aldrich, St. Louis, MO, United States) and 5-iodo-2′-deoxyuridine (IdU; cat. no. I7125, Sigma-Aldrich) were dissolved at a concentration of 15 mg/mL in sterile 0.9% NaCl solution. To assess cell survival, all rats were treated with BrdU at a dose of 50 mg/kg (i.p.) for five consecutive days during the third week of experimentation (Rivera et al., 2015). To assess cell proliferation, IdU were administered at a dose of 57.65 mg/kg (i.p.) for five consecutive days after the fifth week of experimentation (Figure 1).

### Y-Maze Test

The effect of ethanol alone and combined with THC and/or OEA on short-term memory was evaluated using a Y-maze during the fifth week of experimentation. The maze has three interconnected closed arms, each one of them is 120° from the adjacent. The animal was placed into the middle of the maze and was allowed to freely explore the maze for 5 min. The movement of the animal inside the Y-maze was recorded by an overhead camera. The arms were thoroughly cleaned with a disinfectant solution with 70% ethanol and drying napkins to eliminate any residual odors of previous rats. Tests were performed 10 min after cleaning and 30 min after vehicle or OEA administrations. The number of arm entries were counted and acted as a marker of locomotor activity. The number of spontaneous alternations, as defined by entry into three different arms in sequence (triad), serves as a measure of short-term working memory. Percentage of spontaneous alternations was calculated from the number of triads and arm entries using the following equation: Y = number of triads / (total number of arm entries-2) × 100 (Nookala et al., 2018).

### Sample Collection

Previous to sacrifice, all animals were intraperitoneally anesthetized (sodium pentobarbital, 50 mg/kg body weight) in a room separate from the other experimental animals.

The first batch of animals (*n* = 8/group) were sacrificed by decapitation and blood samples (*n* = 8/group) were briefly collected into tubes containing EDTA-2Na (1 mg/mL blood) and centrifuged (1600 *g* for 10 min, 4°C). Plasma samples were then frozen and stored at −80°C for hormonal analysis. The brains were also collected, snap-frozen and stored at −80°C. These brains were then prepared on dry ice to obtain 1-mm-thick sections by using razor blades and a rat brain slicer matrix. The dorsal hippocampus was precisely removed from −2.16 to −4.20 mm of Bregma levels (Paxinos and Watson, 2007) with fine surgical instruments. Hippocampal samples were weighed and stored at −80°C until they were used for real-time quantitative reverse transcription polymerase chain reaction (RT-qPCR) and Western Blot analyses.

A second batch of animals (*n* = 8/group) were perfused transcardially with 4% formaldehyde in 0.1 M phosphate buffer (PB) and the brains were dissected out and kept in the same fixative solution overnight at 4°C. The brains were then cut into 30-μm-thick coronal sections by using a sliding microtome (Leica VT1000S) and divided in 12 parallel series. Sections were stored at 4°C in PB with 0.002% (*w*/*v*) sodium azide until they were used for immunostaining.

### Enzyme-Linked Immunoassay Analysis

Plasma levels of BDNF were determined using the enzyme-linked immunosorbent assay (ELISA) Invitrogen^TM^ BDNF Rat ELISA Kit (#ERBDNF, Thermo Fisher Scientific, United States), following the manufacturer’s instructions. To perform the ELISA protocol in rat samples, we used 25 μL of plasma. A calibration curve and internal controls were included.

### RNA Isolation and RT-qPCR Analysis

We performed RNA isolation, reverse transcription and RT-qPCR (TaqMan, Applied Biosystem, Carlsbad, CA, United States) as described previously (Rivera et al., 2015) using specific sets of primer probes (see Supplementary Table S1). Briefly, hippocampal samples were homogenized on ice and RNA was extracted following Trizol^®^ method according to the manufacture’s instruction (Gibco-BRL Life Technologies, Baltimore, MD, United States). RNA samples were isolated with RNeasy minElute cleanup-kit including digestion with DNase I column (Qiagen, Hilden, Germany). After reverse transcript reaction from 1 μg of mRNA, RT-qPCR was performed in a CFX96TM Real-Time PCR Detection System (Bio-Rad, Hercules, CA, United States) and the FAM dye label format for the TaqMan^®^ Gene Expression Assays (Thermo Fisher Scientific, United States). Melting curve analysis was performed to ensure that only a single product was amplified. Ct values were normalized in relation to *Actb* levels.

### Western Blot Analysis

Western blotting was performed as described previously (Silva-Peña et al., 2018). An appropriate combination of primary and secondary HRP-conjugated antibodies was used: Phospho-(Thr202/Tyr204)-p44/42 MAPK (ERK1/2) rabbit polyclonal antibody (1:1,000, Cell Signaling, #9101), p44/42 MAPK (ERK1/2) rabbit monoclonal antibody (1:1,000, Cell Signaling, #4695), phospho-(Thr308)-AKT1/2/3 rabbit polyclonal antibody (1:1000, Santa Cruz, #sc-16646-R), AKT1/2/3 rabbit polyclonal antibody (1:1000, Santa Cruz, #sc-8312), adaptin γ mouse antibody (1:1,000, BD Biosciences, #610385) and HRP-conjugated anti-rabbit or anti-mouse IgG (H+L) secondary antibodies (1:10,000, Promega, Madison, WI, United States). The membranes were incubated for 1 min with the Western Blotting Luminol Reagent kit (Santa Cruz, CA, United States), and the specific protein bands were visualized and quantified by chemiluminescence using a ChemiDoc^TM^ MP Imaging System (Bio-Rad). Western blots showed that each primary antibody detected a protein of the expected molecular size. The protein intensity was quantified with the image processing software ImageJ (Rasband, W.S., ImageJ, United States, NIH^1^, 1997–2012). The results were expressed as the protein/adaptin γ and phosphorylated/total protein ratios.

### Immunohistochemistry

Free-floating coronal sections from −2.16 to –4.20 Bregma levels (hippocampus) of each parallel series were selected for each immunohistochemistry (Rivera et al., 2015). Sections were incubated overnight in their respective following diluted primary antibodies at 4°C: mouse anti-BrdU (1:2,000; Hybridoma Bank, Iowa City, IA, United States; ref. G3G4), mouse anti-IdU (1:2000; Hybridoma Bank, ref. 32D8.D9) (Rivera et al., 2013). Sections were incubated in the appropriate secondary antibody biotinylated donkey anti-rabbit IgG (1:500; Sigma-Aldrich, #RPN1004) for 90 min The sections were incubated in ExtrAvidin peroxidase (Sigma-Aldrich) diluted 1:2000 in darkness at room temperature for 1 h. Immunolabeling was revealed with 0.05% diaminobenzidine (DAB, Sigma-Aldrich), 0.05% nickel ammonium sulfate and 0.03% H_2_O_2_ in PB-saline.

### Stereological Cell Quantification

5-bromo-2′-deoxyuridine and IdU-immunoreactive (+) cell nuclei that came into focus were manually counted using a standard optical microscope with the 40 × objective (Nikon Instruments Europe B.V., Amstelveen, Netherlands) coupled to the NIS-Elements Imaging Software 3.00 (Nikon). Brain structure analyzed consisted of approximately 8 coronal sections (from −2.16 to −4.20 mm Bregma levels), which resulted in one of every eight equidistant sections (one representative section for each 240 μm) according to the rostro-caudal extent. Estimations of the number of positive cells in the hippocampal neurogenic niche SGZ in both hemispheres were calculated per sections (30 μm deep), according to a rat brain atlas and cytoarchitectonic criteria (Paxinos and Watson, 2007). Quantification was expressed as the average number of positive cells per section for each experimental group.

### Statistical Analysis

**FIGURE 2:**
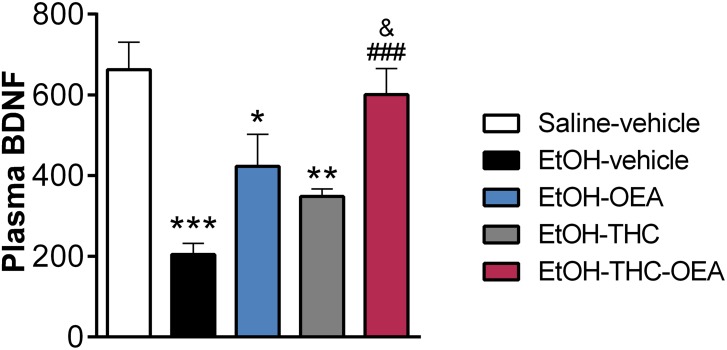
Circulating concentrations of BDNF. Bars represent the mean ± SEM (*n* = 8/group). Tukey: ^∗^*p* < 0.05, ^∗∗^*p* < 0.01, ^∗∗∗^*p* < 0.001 vs. saline-vehicle; ^###^*p* < 0.001 vs. EtOH-vehicle; ^&^*p* < 0.05 vs. EtOH-THC.

**FIGURE 3:**
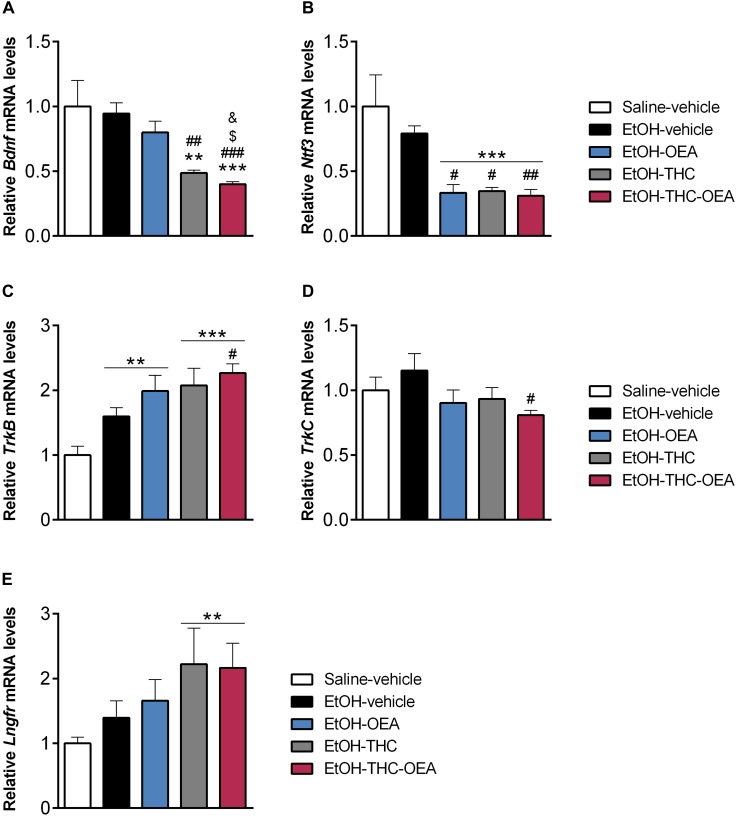
Relative mRNA levels of the neurotrophic factors *Bdnf* **(A)** and *Ntf3* **(B)**, and its receptors *TrkB* **(C)**, *TrkC* **(D)**, and *Lngfr* **(E)** in the hippocampus. Bars represent the mean ± SEM (*n* = 7/group). Tukey **(A–C)** or simple effect analysis **(D,E)**: ^∗∗^*p* < 0.01, ^∗∗∗^*p* < 0.001 vs. saline-vehicle, ^#^*p* < 0.05, ^##^*p* < 0.01, ^###^*p* < 0.001 vs. EtOH-vehicle, and ^&^*p* < 0.05 vs. EtOH-THC.

**FIGURE 4:**
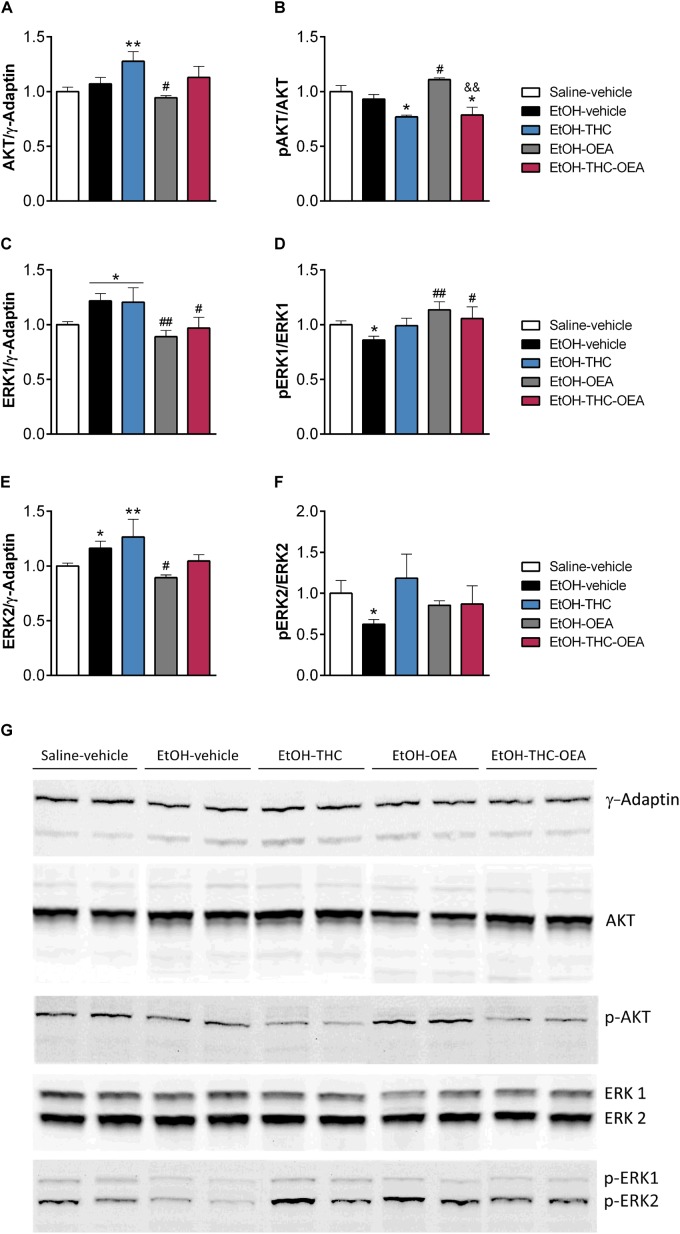
Relative protein levels of AKT **(A)**, phospho-AKT **(B)**, ERK1 **(C)**, phospho-ERK1 **(D)**, ERK2 **(E)** and phospho-ERK2 **(F)** in the hippocampus as well as representative immunoblots **(G)**. Bars represent the mean ± SEM (*n* = 4/group). Tukey **(A–E)** or simple effect analysis **(F)**: ^∗^*p* < 0.05, ^∗∗^*p* < 0.01 vs. saline-vehicle, ^#^*p* < 0.05, ^##^*p* < 0.01 vs. EtOH-vehicle, and ^&&^*p* < 0.01 vs. EtOH-THC.

**FIGURE 5:**
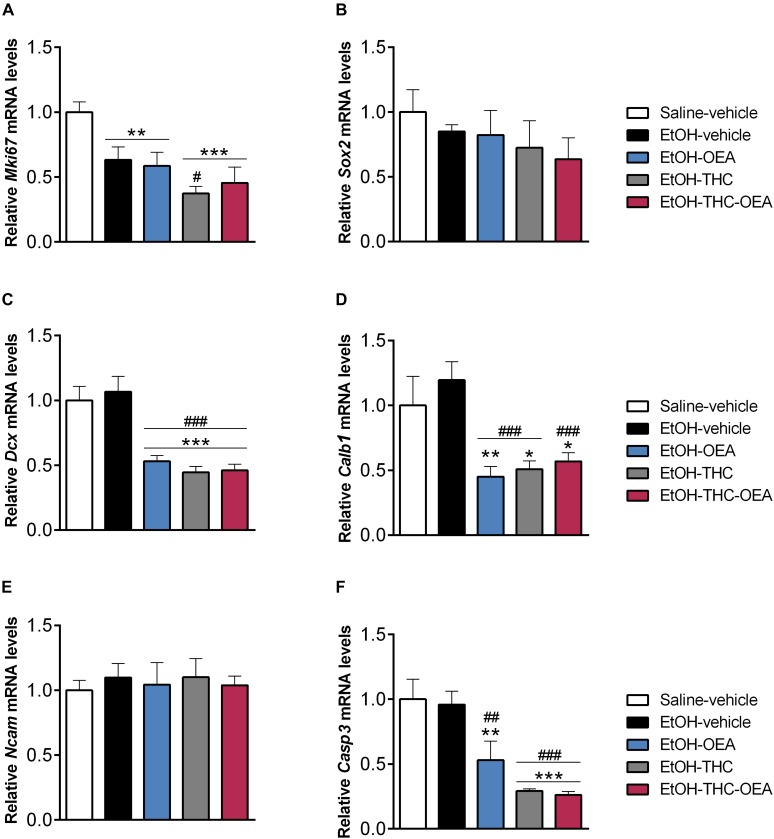
Relative mRNA levels of the neurogenic factors *Mki67* **(A)**, *Sox-2* **(B)**, *Dcx* **(C)**, *Calb1* **(D)** and *Ncam* **(E)**, and the apoptotic protease *Casp3* **(F)** in the hippocampus. Bars represent the mean ± SEM (*n* = 7/group). Tukey **(A,C,D,F)** or simple effect analysis **(B,E)**: ^∗^*p* < 0.05, ^∗∗^*p* < 0.01, ^∗∗∗^*p* < 0.001 vs. saline-vehicle; ^##^*p* < 0.01, ^###^*p* < 0.001 vs. EtOH-vehicle.

**FIGURE 6:**
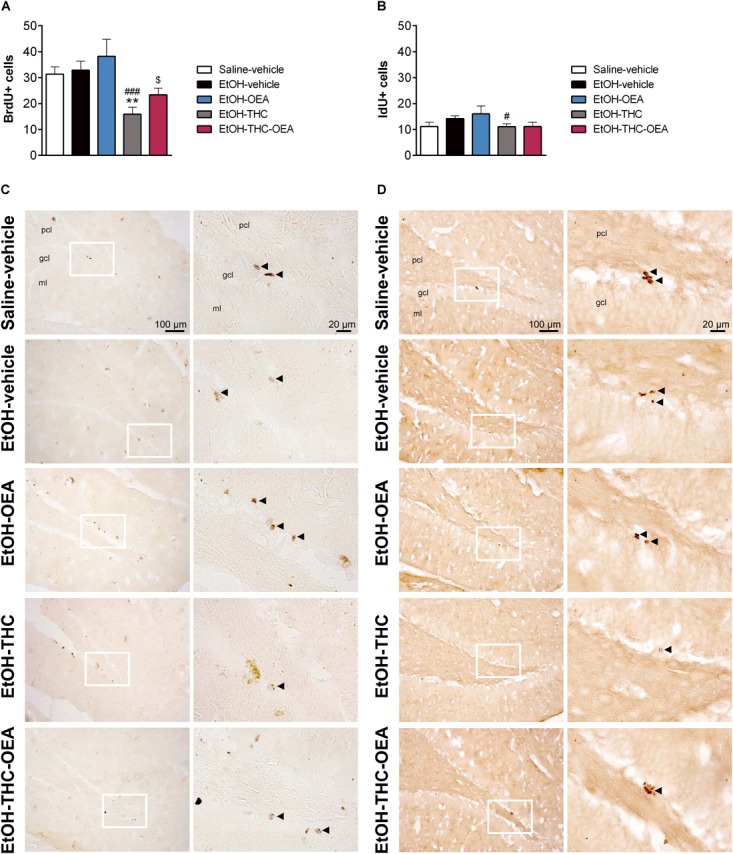
Newborn cell survival **(A)** and neural stem cell proliferation **(B)** respectively, assessed by the number of BrdU and IdU-immunoreactive (+) cells in the SGZ of the dentate gyrus. Low and high-resolution photomicrographs of representative images showing BrdU+ cells **(C)** and IdU+ cells, and **(D)**. Arrowheads indicate labeled nuclei. Bars represent the mean ± SEM (*n* = 7–8/group). Tukey **(A)** or simple effect analysis **(B)**: ^∗∗^*p* < 0.01 vs. saline-vehicle and ^#^*p* < 0.05, ^###^*p* < 0.001 vs. EtOH-vehicle.

**FIGURE 7:**
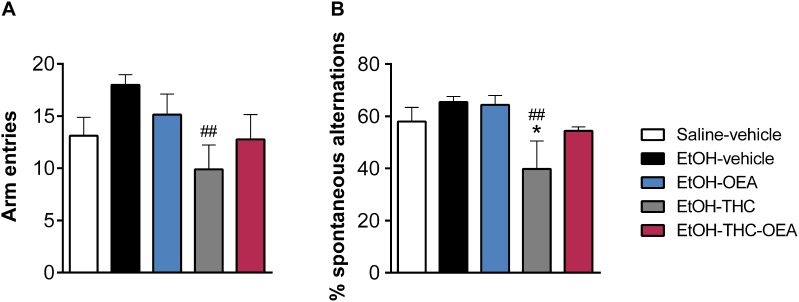
Y-maze test showing the number of arm entries **(A)** and percentage of spontaneous alternations **(B)**. Bars represent the mean ± SEM (*n* = 8/group). Tukey: ^∗^*p* < 0.05 vs. saline-vehicle and ^##^*p* < 0.01 vs. EtOH-vehicle.

Comparisons of data were carried out using IBM SPSS Statistical version 22 software (IBM, Armonk, NY, United States) and GraphPad Prism version 6 software (GraphPad Software, San Diego, CA, United States). Data are expressed as mean ± SEM for up to 8 determinations per experimental group (see legends in Figure 2–7). The Kolmogorov-Smirnov normality test, along with the Levene homoscedasticity test, were used to verify Gaussian distribution. Statistical analyses were performed using one-way analysis of variance (ANOVA) and two-way ANOVA (factor as OEA and THC), and Tuckey’s test corrected for multiple comparison or simple effect analysis when appropriate. A *p*-value less than 0.05 was considered statistically significant.

## Results

### The Combined Administration of OEA and THC During Adolescence Restored the Ethanol-Induced Decreases in the Plasma Levels of BDNF

Significant changes in the circulating levels of BDNF were found (*F*_4,35_ = 10.80; *p* < 0.0001). Plasma levels of BDNF were decreased in the rats exposed to ethanol compared to those of saline-vehicle ones (^∗∗∗^*p* < 0.001) (Figure 2). Two-way ANOVA did not indicate any significant interaction between factors (OEA and THC), but showed main effects of OEA (*F*_1,28_ = 16.88; *p* = 0.0003) and THC (*F*_1,28_ = 7.874; *p* = 0.009) on the plasma levels of BDNF. OEA or THC alone partially reversed ethanol-induced BDNF reduction in plasma, because significant differences were still found compared to saline-vehicle ones (^∗^*p* < 0.05, ^∗∗^*p* < 0.01, respectively). The combined administration of OEA and THC completely restored the decrease in the plasma levels of BDNF observed in rats exposed to ethanol (^###^*p* < 0.001) and THC/ethanol-exposed rats (^&^*p* < 0.05) (Figure 2). OEA at a dose of 5 mg/kg/day administered for 6 consecutive days did not modify significantly the plasma levels of BDNF (Supplementary Figure S1).

### OEA Decreases *Bdnf*, *Ntf3*, and *TrkC*, and Increases *TrkB* in the Hippocampi of Rats Exposed to Ethanol Binges and THC During Adolescence

Statistical analysis indicated significant differences in the hippocampal mRNA levels of *Bdnf*, *Ntf3*, and *TrkB* (*F*_4,30_ > 6.59; *p* < 0.0006), but not *TrkC* and *Lngfr*. The hippocampi of rats exposed to ethanol had higher mRNA levels of *TrkB* compared to those of saline-vehicle ones (^∗∗^*p* < 0.01) (Figure 3A–E). Two-way ANOVA indicated significant interaction between factors (OEA and THC) in the mRNA levels of *Ntf3* (*F*_1,24_ = 16.44; *p* < 0.0001). A main effect of OEA on the mRNA levels of *Ntf3* was found (*F*_1,24_ = 22.49; *p* < 0.0001). Main effects of THC on the mRNA levels of *Bdnf* (*F*_1,24_ = 41.08; *p* < 0.0001) and *Ntf3* (*F*_1,24_ = 20.11; *p* < 0.0001) were also observed. OEA decreased hippocampal mRNA levels of *Ntf3* in ethanol-exposed rats compared to those of ethanol-vehicle rats (^#^*p* < 0.05) and saline-vehicle rats (^∗∗∗^*p* < 0.001) (Figure 3B). THC decreased hippocampal mRNA levels of *Bdnf* and *Ntf3* in ethanol-exposed rats compared to those of ethanol-vehicle rats (^#^*p* < 0.05, ^##^*p* < 0.01) and saline-vehicle rats (^∗∗^*p* < 0.01, ^∗∗∗^*p* < 0.001) (Figure 3A,B). OEA in THC/ethanol-exposed rats also decreased hippocampal mRNA levels of *Bdnf* and *Ntf3*, as well as *TrkC*, but specifically increased mRNA levels of *TrkB* compared to those of ethanol-vehicle rats (^#^*p* < 0.05, ^##^*p* < 0.01, ^###^*p* < 0.001) (Figure 3A–D). The hippocampi of THC/ethanol-exposed rats with and without OEA administration had higher mRNA levels of *Lngfr* compared to those of saline-vehicle rats (^∗∗^*p* < 0.01) (Figure 3E).

### OEA Increases the Phosphorylation of AKT and ERK1 in the Hippocampi of Rats Exposed to Ethanol Binges During Adolescence

Statistical analysis indicated significant changes in the protein levels of total and phosphorylated AKT (*F*_4,21_ > 3.60; *p* < 0.026), total and phosphorylated ERK1 (*F*_4,21_ > 3.16; *p* < 0.040), and total ERK2 (*F*_4,21_ = 4.14; *p* < 0.015) in the hippocampus. Specifically, the hippocampi of rats exposed to ethanol had higher total protein levels, but lower phosphorylated protein levels of ERK1 and ERK2 compared to those of saline-vehicle ones (^∗^*p* < 0.05) (Figure 4A–G). Two-way ANOVA did not indicate any significant interaction between factors (OEA and THC). Main effects of THC on the total (*F*_1,14_ = 7.14; *p* = 0.023) and phosphorylated (*F*_1,14_ = 20.14; *p* = 0.001) protein levels of AKT were observed. Main effects of OEA on the total protein levels of ERK1 (*F*_1,14_ = 9.58; *p* = 0.011) and ERK2 (*F*_1,14_ = 7.73; *p* = 0.019), and phosphorylated protein levels of ERK1 (*F*_1,14_ = 6.29; *p* = 0.031) were found. THC increased total protein levels of AKT, ERK1 and ERK2 (Figure 4A,C,E), but decreased AKT phosphorylation (Figure 4B) in ethanol-exposed rats compared to those of saline-vehicle rats (^∗^*p* < 0.05, ^∗∗^*p* < 0.01). OEA decreased total protein levels of AKT, ERK1, and ERK2 in ethanol-exposed rats compared to those of ethanol-vehicle rats (^#^*p* < 0.05, ^##^*p* < 0.01) (Figure 4A,C,E). OEA increased the phosphorylated protein levels of AKT and ERK1 in ethanol-exposed rats compared to those of ethanol-vehicle rats (^#^*p* < 0.05, ^##^*p* < 0.01) (Figure 4B,D). OEA administered in THC/ethanol-exposed rats did not increase AKT phosphorylation as significant differences were found compared to OEA/ethanol-exposed rats (^&&^*p* < 0.01) (Figure 4B). Contrary, the increased ERK1 phosphorylation was conserved when OEA was administered in THC/ethanol-exposed rats (^#^*p* < 0.05) (Figure 4D).

### The Combined Administration of OEA and THC Decreases *Mki67*, *Dcx*, *Calb1*, and *Casp3* in the Hippocampi of Rats Exposed to Ethanol Binges During Adolescence

Statistical analysis indicated significant differences in the hippocampal mRNA levels of *Mki67*, *Dcx*, *Calb1*, and *Casp3* (*F*_4,30_ > 6.37; *p* < 0.0008), but not *Sox2* and *Ncam*. Specifically, the hippocampi of rats exposed to ethanol had lower mRNA levels of the cellular marker for proliferation *Mki67* compared to those of saline-vehicle rats (^∗∗^*p* < 0.01) (Figure 5A). Two-way ANOVA indicated significant interaction between factors (OEA and THC) in the mRNA levels of *Dcx* (*F*_1,24_ = 12.54; *p* = 0.0017), *Calb1* (*F*_1,24_ = 15.88; *p* = 0.0005), and *Casp3* (*F*_1,24_ = 4.81; *p* = 0.038). Main effects of OEA and THC on the mRNA levels of *Dcx* (*F*_1,24_ > 11.25; *p* < 0.002), *Calb1* (*F*_1,24_ > 7.87; *p* < 0.009) and *Casp3* (*F*_1,24_ > 6.40; *p* < 0.01) were also observed. The administration of OEA and THC alone decreased mRNA levels of *Dcx*, *Calb1*, and *Casp3* in ethanol-exposed rats compared to those of saline-vehicle rats (^∗^*p* < 0.05, ^∗∗^*p* < 0.01, ^∗∗∗^*p* < 0.001) and ethanol-vehicle rats (^##^*p* < 0.01/0.001, ^###^*p* < 0.001) (Figure 5C–F). Specifically, simple effect analysis indicated that THC alone decreased mRNA levels of *Mk67* in ethanol-exposed rats (^#^*p* < 0.05), but this effect was erased when OEA was also administered (Figure 5A). No significant changes in mRNA levels of *Sox2* and *Ncam* were observed (Figure 5B,E).

### OEA Modulates the THC-Related Decreases in Both Neural Stem Cell Proliferation and Newborn Cell Survival in the SGZ of Rats Exposed to Ethanol Binges During Adolescence

Statistical analysis indicated significant changes in the number of BrdU+ cells (*F*_4,35_ = 5.70; *p* < 0.0013), but not the number of IdU+ cells. Specifically, ethanol binges did not produce any effect on the number of BrdU+ cells (newborn cell survival) and the number of IdU+ cells (cell proliferation) in the SGZ of the dentate gyrus (Figure 6). Two-way ANOVA did not indicate any significant interaction between factors (OEA and THC). Main effects of THC, but not OEA, on the number of BrdU+ cells (*F*_1,8_ = 11.84; *p* < 0.01) and IdU+ cells (*F*_1,8_ = 10.53; *p* = 0.011) were observed. OEA alone did not modify the number of BrdU+ and IdU+ cells in the SGZ of ethanol-exposed rats. THC reduced the number of BrdU+ and IdU+ cells in ethanol-exposed rats compared to those of saline-vehicle rats (^∗∗^*p* < 0.01) and ethanol-vehicle rats (^###^*p* < 0.001) (Figure 6A,B). However, these effects of THC on SGZ cell proliferation and survival in ethanol-exposed rat were modulated when OEA was also administered.

### OEA Blocked Short-Term Spatial Memory Impairment in Rats Exposed to THC and Ethanol Binges During Adolescence

Statistical analysis indicated significant differences in the ratio between series and entries (percentage of spontaneous alteration) in the Y-maze (*F*_4,35_ = 3.10; *p* = 0.026), but not the number of arm entries (*F*_4,35_ = 2.26; *p* = 0.058), used as an index of activity. Specifically, ethanol binges did not produce any effect on spontaneous alternation or activity (arm entries) in the Y-maze (Figure 7). Two-way ANOVA did not indicate any significant interaction between factors (OEA and THC). Main effects of THC, but not OEA, on the number of arm entries (*F*_1,26_ = 6.46; *p* = 0.016) and the percentage of spontaneous alteration (*F*_1,26_ = 6.03; *p* = 0.02) were observed. THC affected both memory (reduced spontaneous alternation) and activity (arm entries) in ethanol-exposed rats (^##^*p* < 0.01) (Figure 7A,B). This memory and motor impairment induced by the combination of THC and ethanol was blocked by OEA (Figure 7A,B).

## Discussion

**FIGURE 8:**
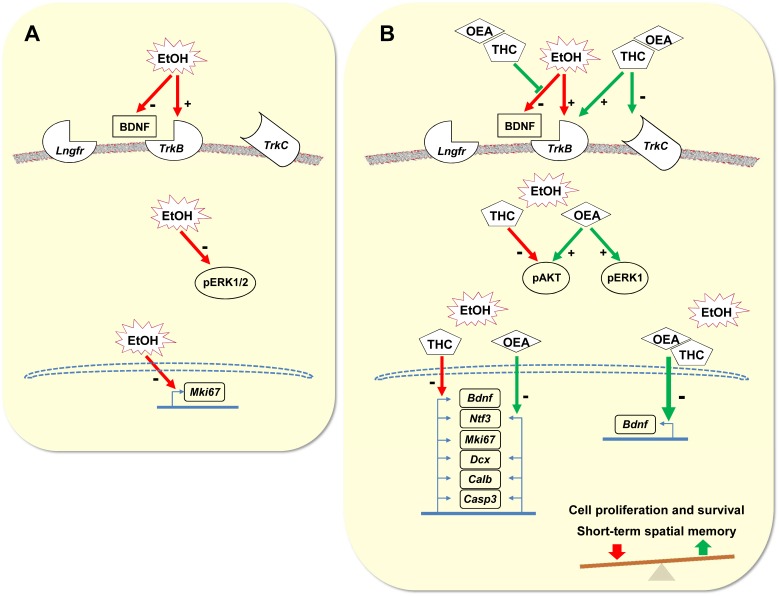
Schematic representation that hypothesizes the effects of ethanol **(A)**, THC and/or OEA **(B)** on neurotrophic signaling pathway via AKT and ERK1/2, and its neurogenic response in the hippocampus.

In the present study we evaluated the effects of OEA treatment (10 mg/kg, i.p.), 48 h after acute administration of THC (5 mg/kg, i.p.) and/or ethanol binge (3 g/kg, i.g.), on the mRNA levels of main components of the neurotrophic (BDNF and NT3) system, the phosphorylation of intracellular protein kinases (AKT, ERK), and the cell proliferation, maturation and survival in the dorsal hippocampus. The Y-Maze test was also tested as a behavioral assessment tool for short-term spatial memory. The main findings are as follows (see Figure 8 for summary): (1) The regimen of ethanol binges decreased the plasma concentrations of BDNF, the phosphorylated levels of the MAP kinases ERK1/2, the mRNA levels of the neurotrophic receptor *TrkB* and the cellular factor for proliferation *Mki67* in the hippocampus (Figure 8A), but did not affect other factors related to neurogenesis or spatial memory, (2) The combination of THC and ethanol reduced short-term spatial memory, the mRNA levels of *Bdnf* in the hippocampus, and the cell proliferation (*Mki67* and IdU+ cell population) and survival (*Casp3* and BrdU+ cell population) in the dorsal hippocampus, (3) The repeated administration of OEA in the ethanol-exposed rats modulated the plasma levels of BDNF, and increased the hippocampal levels of phospho-AKT and phospho-ERK1, key signaling regulators of neurogenesis and cell survival (Figure 8B), (4) Both OEA and THC produced similar effects on the mRNA levels of neurotrophic (*Ntf3*), maturation (*Dcx*, *Calb1*) and pro-apoptotic (*Casp3*) factors in the hippocampus of ethanol-exposed rats, (5) Interestingly, the repeated administration of OEA in rats previously exposed to both ethanol and THC normalized the plasma levels of BDNF, enhanced the effects on the hippocampal mRNA levels of *Bdnf* and the neurotrophic receptors *TrkB* and *TrkC*, and sustained the phosphorylated levels of ERK1, and (6) These last effects were likely associated with a recovery of short-term spatial memory and newborn cell survival in the SGZ of the dentate gyrus (Figure 8B).

We have recently demonstrated that the continuous exposure of ethanol in a 4-bottle choice paradigm affects neurotrophin signaling pathway through a deactivation of the neurogenic regulator MAP kinase ERK2 and a decrease in mRNA levels of the neurogenic factors *Mki67*, *Sox2*, *Dcx*, *Ncam1*, and *Calb1* (Silva-Peña et al., 2018). Nevertheless, the concrete effect of ethanol on neurotrophic factors remains unclear, and different associations between BDNF and ethanol consumption have been previously described (Joe et al., 2007; Costa et al., 2011; Reynolds et al., 2015; Geoffroy and Noble, 2017). In this regard, previous studies indicated that a decrease in BDNF levels in plasma was associated with ethanol consumption (García-Marchena et al., 2017b; Silva-Peña et al., 2018). This effect was also linked to withdrawal severity (Heberlein et al., 2010). In the present study, we go further into this hypothesis as we described a decrease in BDNF levels in the plasma of rats exposed to ethanol binges (3 g/kg) once a week for 5 weeks during adolescence. This finding is relevant for establishing BDNF as a biomarker of single weekly alcohol consumption during adolescence despite not affecting neither cognition nor neurogenesis.

We also examined the effect of ethanol binges together with the administration of THC (5 mg/kg), a principal psychoactive component of cannabis that activates CB1 and CB2 receptors. Cannabis is the most widely used illicit drug, and the disruption of learning and memory are commonly reported consequences of cannabis use. In agreement with our results in ethanol-exposed rats, spatial memory impairment by THC has been demonstrated in adolescent animals (Steel et al., 2011; Segal-Gavish et al., 2017) and healthy humans (D’Souza et al., 2009). Molecular mechanisms underlying behavioral alteration by cannabis are not completely described, although the importance of adaptive changes in neuroplasticity (synaptic number and strength) and neurogenesis during learning are accepted (Steel et al., 2014). Endocannabinoid system regulates several physiological processes such as anxiety (Ahn et al., 2008), nociception (Pertwee, 2001) and memory (Marsicano et al., 2002). Systemic and intra-hippocampal administration of CB1 receptors agonists, including THC (5.6 mg/kg, i.p.), impair hippocampal-dependent memory/learning tasks (Wegener et al., 2008; Wise et al., 2009; Hasanein and Teimuri Far, 2015). Moreover, intra-hippocampal administration of the CB1 receptor antagonist rimonabant (0.06 μg/rat) completely attenuated the memory disruptive effects of cannabinoids (Wise et al., 2009). This tendency is consistent with our study because THC induced short-term memory impairment assessed by lower spatial alternations. However, the effects of cannabinoids on plasticity activity of the neuron do not necessary follow simple patterns, particularly when memory processes are involved (Abush and Akirav, 2010). In fact, a recent dose-response study (Suliman et al., 2018) reported that THC administered in rats at a dose of 1.5 mg/kg improved cognitive functions and enhanced the markers involved in hippocampal neurogenesis including DCX and BDNF. These effects were not observed when THC was administered at doses of 0.5 and 3 mg/kg (Suliman et al., 2018), suggesting a biphasic (impairing or enhancing) role of CB1 receptor activation in both adult neurogenesis and memory activities (Calabrese and Rubio-Casillas, 2018). Other studies have shown that THC prevents neurodegenerative processes occurring in animal models of Alzheimer’s disease, protects from inflammation-induced cognitive damage, and restores memory and cognitive function in old mice (Martín-Moreno et al., 2012; Aso et al., 2015). CB2 receptor was also related to memory and synaptic plasticity (García-Gutiérrez et al., 2013; Li and Kim, 2016). Activation of CB2 receptors by the administration of different selective CB2 receptor agonists (O-1966 and MDA7) reversed amyloid-induced memory deficiency and improves memory retention following stroke in mice (Wu et al., 2013; Ronca et al., 2015). So, we cannot discard that the biphasic effect of THC on neural plasticity could be linked to a putative positive/negative balance between CB1/CB2 receptor activation. Regarding our results, we propose that THC at a dose of 5 mg/kg likely facilitates the expected deleterious effects of alcohol on hippocampal neurogenesis and spatial memory.

Oleoylethanolamide is a satiety factor capable of controlling contextual memories associated with alcohol relapse (Bilbao et al., 2016). In addition, alcohol withdrawal symptoms are supported by the decrease in OEA levels and its adaptive nature as a homeostatic signal (Bilbao et al., 2016). Interestingly, OEA injection (5 and 20 mg/kg) at the beginning of withdrawal (when OEA levels are dropping) produced a lower severity of the withdrawal symptoms (Bilbao et al., 2016). These results support previous clinical studies suggesting that OEA levels are altered in alcohol-dependence during abstinence and might be a potential marker for predicting length of alcohol abstinence (García-Marchena et al., 2017a). Human genetic reports also described an association between AUD and polymorphisms/mutation of the gene encoding the NAE-hydrolyzing enzyme fatty-acid amide hydrolase (FAAH) (Sipe et al., 2002). Inhibition of FAAH, the enzyme responsible for the degradation of the NAEs anandamide and OEA (Cravatt et al., 1996), enhances memory and does not induce the adverse effects of CB1 receptor agonists (Hasanein and Teimuri Far, 2015; Rivera et al., 2018). Moreover, inhibition of FAAH promotes memory acquisition through OEA and PPARα activation (Mazzola et al., 2009). Our findings reinforce the hypothesis that repeated treatment of OEA can exert functional recovery of THC-related cognitive impairments and neuroprotective effects via triggering of adult neurogenesis in the hippocampi of ethanol-exposed rats during adolescence.

Despite the association between OEA and BDNF has been barely studied, the chronic treatment with OEA markedly improves spatial cognitive deficits through enhancing neurogenesis and BDNF expression in the hippocampus after acute cerebral ischemic injury (Yang et al., 2015). Several studies reported that THC increases BDNF levels in the plasma of rats (Suliman et al., 2018) and the serum of healthy humans (D’Souza et al., 2009). In our study, plasma BDNF concentrations were decreased after ethanol binges. Interestingly, while THC and OEA slightly or partially blocked the ethanol effect when they were separately administered, the combination of both drugs in the ethanol-exposed rats induced a synergistic effect that completely equalized BDNF levels to saline-vehicle group. The molecular mechanisms involved in the ethanol-induced decrease of circulating BDNF remain to be elucidated but reinforces the notion of BDNF as a biomarker of alcohol consumption.

The relevance of these findings was further explored when we analyzed hippocampal expression of neurotrophins, their receptors, molecular signaling, and transcriptional response. Interestingly, THC decreased the mRNA levels of *Bdnf* and *Ntf3* in the hippocampus. Concomitant OEA and THC did not restore neurotrophin expression but, synergistically, increased the mRNA levels of the receptor binding BDNF *TrkB* and decreased the mRNA levels of receptor binding NT-3 *TrkC*. Further studies are necessary to explain the apparent contradiction between the effects of THC and OEA on the hippocampal expression of BDNF and its receptor TrkB. Besides, synergistic reduction in *Ntf3* and *TrkC* mRNA levels by THC and OEA suggest a relevant impact on NT-3 signaling system in a context of ethanol binges. This hypothesis is consistent with the intracellular signaling in the hippocampi of ethanol-exposed rats. The decreased levels of ERK1 phosphorylation induced by ethanol was specifically counteracted by OEA. We propose that this effect may be related to a sensitization of BDNF-TRKB signaling. Moreover, the increased levels of AKT phosphorylation induced by OEA agree with an activation of this signaling pathway (please, see Figure 8B for schematic summary). Regarding the neurogenic response, THC specifically enhanced the ethanol-related reduction in mRNA levels of the cellular factor for proliferation *Mki67*, an effect that was in agreement with the lower number of IdU+ cells in the SGZ. Similar effects of THC and OEA were found on the factors maturing neurons *Dcx* and *Calb1*. Besides, hippocampal mRNA levels of *Casp3*, a neural marker of apoptosis, was also decreased by both OEA and THC. Interestingly, OEA blocked the lower number of SGZ BrdU+ cells induced by THC. These last results suggest that both OEA and THC may abrogate a cell death response in the hippocampus under an alcoholic context, but only OEA may amplify this anti-apoptotic signal into the hippocampal neurogenic niche.

## Conclusion

In conclusion, ethanol binge drinking during adolescence impairs circulating BDNF concentrations and ERK signaling in the hippocampus. THC associated with ethanol specifically induced a deleterious effect on both short-term memory and in a number of neurotrophic (*Bdnf*, *Ntf3*) and neurogenic (*Mki67*, *Dcx*, *Calb1*) factors in the hippocampus, a region widely related to synaptic plasticity of the neuron. Administration of OEA alone or in combination with THC in ethanol-exposed rats restored ethanol-related BDNF deficiency in plasma, increased the expression of the BDNF receptor *TrkB* and intracellular signaling (AKT and ERK1 phosphorylation), and sustained a reduced expression of maturation (*Dcx* and *Calb1*) and pro-apoptotic (*Casp3*) responses in the hippocampus. Finally, OEA may be related to a likely positive effect on newborn cell survival in the hippocampus and short-term spatial memory. Therefore, OEA and others putative PPARα activators are interesting targets to be further investigated to unveil novel therapeutic strategies approaching cognitive impairment and pathologies related to AUD.

## Limitations and Future Perspective

This study examined the single effects of THC and OEA, and their interaction, on short-term spatial memory and neurogenesis through BDNF/AKT/ERK signaling in the dorsal hippocampus of adolescent rats exposed to ethanol binge drinking. It was not intended to evaluate the actions of these compounds in normal animals, so we decided to focus only in ethanol-exposed ones. Although our findings support the hypothesis of a protective role of OEA in response to ethanol, we are aware of the limitations of the present study. First, the lack of females rats in the experimental design is an important limitation of the present study. Same-sex representativeness and the effect of sexual dimorphism on reactivity to abuse drugs and treatments should be definitely addressed for all experiments in pre-clinical research (McCullough et al., 2014). Second, the lack of a factorial design that includes naïve-ethanol groups is another important limitation of the present study. Despite main effects and interaction of OEA and THC in rats exposed to ethanol were evaluated by two-way ANOVA, independent effects and interactive effects between drugs and ethanol were not assessed, limiting our findings to the alcohol-exposure context only. Third, the experiments of the present study were designed to mimic the one-day heavy drinking of adolescents and to evaluate vulnerability to ethanol during adolescence (Fabio et al., 2014), so we cannot extrapolate to heavy binge drinking in adults. In addition, the magnitude of the effect cannot be estimated because we used only one dose for OEA or THC. Although drug doses were selected regarding effectiveness based on previous studies (Piomelli et al., 2006; Galan-Rodriguez et al., 2009; Bilbao et al., 2016; Murphy et al., 2017; Antón et al., 2017, 2018), the use of a dose-response design might have offered more information concerning the effects of the drugs tested. Finally, although the amount of ethanol administered with the vehicle solution for THC was small (0.2 g/kg), we must consider the potential induction of biological responses that could interfere with the outcomes measured.

## Ethics Statement

All protocols were approved by the Ethics and Research Committee of Universidad de Málaga (CEUMA, 7-2016-A).

## Author Contributions

FR and JS conceived and designed the study. DS-P, PR, FA, AV, and NG-M acquired the data. DS-P, PR, LR, FP, and AS analyzed and interpreted the data. DS-P and JS drafted the manuscript. FR and JS Reviewed and edited the manuscript. All authors approved the final version of the manuscript.

## Conflict of Interest Statement

The authors declare that the research was conducted in the absence of any commercial or financial relationships that could be construed as a potential conflict of interest.

## Footnotes

1 http://imagej.nih.gov/ij

## Abbreviations

9-THC: delta-9-tetrahydrocannabinol or dronabinol
AKT (pAKT): protein kinase B (phosphorylated)
BDNF/Bdnf: brain-derived neurotrophic factor
BrdU: 5-bromo-2′-deoxyuridine
Calb1: calbindin
Casp3: caspase 3
dcx: doublecortin
ERK1/2 (pERK1/2): extracellular signal-regulated kinases 1/2 or MAP kinases 1/2 (phosphorylated)
gcl: granular cell layer of dentate gyrus
IdU: 5-iodo-2-deoxyuridine
Lngfr: low-affinity nerve growth factor receptor
Mki67: antigen KI-67
ml: molecular layer of dentate gyrus
NCAM: enural cell adhesion molecule
Ntf3: neurotrophin-3
OEA: oleoylethanolamide
PCL: polymorphic cell layer of dentate gyrus
SGZ: subgranular zone of the dentate gyrus
Sox2: sex determining region Y (SRY)-box 2
Str: striatum
TrkB: tropomyosin receptor kinase B
TrkC: tropomyosin receptor kinase C.
