# Supplementary material for: Oleoylethanolamide Modulates BDNF-ERK Signaling and Neurogenesis in the Hippocampi of Rats Exposed to Δ9-THC and Ethanol Binge Drinking During Adolescence
Source: Front Mol Neurosci. 2019 Apr 24;12:96. doi: 10.3389/fnmol.2019.00096 (PMC6491684; doi:10.3389/fnmol.2019.00096)
Supplement: Supplementary file 1 [file Table_1.DOCX]

**Table S1.** List of specific primers used to perform PCR analysis from TaqMan® Gene Expression Assays (ThermoFisher).^1^

| **Gen name** | **Gen code** | **Amplicon length** |
| --- | --- | --- |
| *Actb* | Rn00667869_m1 | 91 |
| *Bdnf* | Rn02531967_s1 | 142 |
| *Ntf3* | Rn00579280_m1 | 105 |
| *Ntrk2 (TrkB)* | Rn01441749_m1 | 73 |
| *Ntrk3 (TrkC)* | Rn00570389_m1 | 63 |
| *Ngfr (Lngfr)* | Rn00561634_m1 | 60 |
| *Mki67* | Rn01451446_m1 | 104 |
| *Sox2* | Rn01286286_g1 | 93 |
| *Dcx* | Rn00670390_m1 | 72 |
| *Ncam1* | Rn01418541_m1 | 59 |
| *Casp3* | Rn00563902_m1 | 93 |
| *Calb1* | Rn00583140_m1 | 69 |

^1^Abbreviations: *Actb*, beta actin; *Bdnf*, brain derived neurotrophic factor; *Ntf3*, neurotrophin 3; *Ntrk2/3* or *TrkB/C*, tropomyosin receptor kinase B/C; *Ngfr* or *Lngfr*, low-affinity nerve growth factor receptor or p75 neurotrophin receptor; *Mki67*, antigen KI-67; *Sox2*, sex determining region Y-box 2; *Dcx*, doublecortin; *Ncam1*, neural cell adhesion molecule; *Casp3,* Caspasa 3; *Calb1*, calbindin.
