# Supplementary material for: Oleoylethanolamide Modulates BDNF-ERK Signaling and Neurogenesis in the Hippocampi of Rats Exposed to Δ9-THC and Ethanol Binge Drinking During Adolescence
Source: Front Mol Neurosci. 2019 Apr 24;12:96. doi: 10.3389/fnmol.2019.00096 (PMC6491684; doi:10.3389/fnmol.2019.00096)
Supplement: Supplementary file 2 [file Data_Sheet_1.pdf]

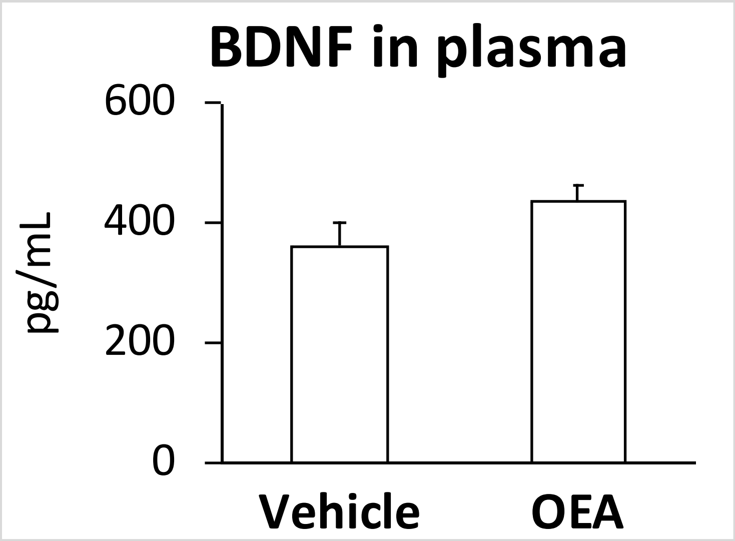

FIGURE S1 | Circulating concentrations of BDNF in naïve rats treated with OEA (5 mg/kg/day) for 6 days. Bars represent the mean + SEM (n = 8/group).
